# Supplementary material for: The Escherichia coli replication initiator DnaA is titrated on the chromosome
Source: Nat Commun. 2025 Aug 21;16:7813. doi: 10.1038/s41467-025-63147-1 (PMC12371093; doi:10.1038/s41467-025-63147-1)
Supplement: Supplementary file 1 — Supplementary Information [file 41467_2025_63147_MOESM1_ESM.pdf]

Supplementary Information

Table S1. List of bacterial strains used in this study, with related genotype and source. *DnaA(n1-n2)* stands for the sequence of the *dnaA* gene comprised between base pair n1 and base pair n2, counting from the G at position 1.

Strain	Characteristics	Source
E. coli MG1655	K-12; F⁻ λ⁻ *rph-1*	ATCC
E. coli Δ3D	MG1655; *strR* Δ*DARS1* Δ*DARS2* Δ*datA*	[1]
E. coli Δ*datA*	MG1655; *strR* Δ*datA*	[1]
E. coli Δ*DARS1*	MG1655; *strR* Δ*DARS1*	[1]
E. coli Δ*DARS2*	MG1655; *strR* Δ*DARS2*	[1]
E. coli DH5α	MG1655; *endA1 glnV44 thi-1 recA1 relA1 gyrA96 deoR nupG* ϕ80d*lacZ*ΔM15 Δ(*lacZYA-argF)U169 hsdR17* (r_K^- m_K^+)	New England Biolabs
E. coli DH5α x pCas9_ampR	DH5a harbouring the pCas9_ampR plasmid	This study
E. coli DH5α x pTarget_dnaA-PAFP	DH5a harbouring one of the four plasmids of the pTarget_dnaA-PAFP series	This study
E. coli MG1655 x pCas9	MG1655 harbouring the pCas9 plasmid	This study
E. coli dnaA-PAmCherry2.1	MG1655; *dnaA*Δ*(259-312)::PAmCherry2.1*	This study
E. coli dnaA-dronpa2	MG1655; *dnaA*Δ*(259-312)::dronpa2*	This study
E. coli dnaA-mEos4b	MG1655; *dnaA*Δ*(259-312)::mEos4b*	This study
E. coli dnaA-mMaple3	MG1655; *dnaA*Δ*(259-312)::mMaple3*	This study
E. coli Δ3D x pCas9_ampR	MG1655 harbouring the pCas9_ampR plasmid; *strR* Δ*DARS1* Δ*DARS2* Δ*datA*	This study
E. coli Δ3D *dnaA-PAmCherry2.1*	MG1655; *strR* Δ*datA* Δ*DARS1* Δ*DARS2* *dnaA*Δ*(259-312)::PAmCherry2.1*	This study
E. coli Δ*datA dnaA-PAmCherry2.1*	MG1655; Δ*datA dnaA*Δ*(259-312)::PAmCherry2.1*	This study
E. coli Δ*DARS1 dnaA-PAmCherry2.1*	MG1655; Δ*DARS1 dnaA*Δ*(259-312)::PAmCherry2.1*	This study
E. coli Δ*DARS2 dnaA-PAmCherry2.1*	MG1655; Δ*DARS2 dnaA*Δ*(259-312)::PAmCherry2.1*	This study
E. coli MG1655 x pDnaA211-PamCherry2.1	MG1655 harbouring the pDnaA211-PAmCherry2.1 plasmid	This study
E. coli MG1655 x pdnaAp-LacZ	*E. coli* MG1655 harbouring the pdnaAp-LacZ plasmid	This study
E. coli dnaA-PAmCherry2.1 x pdnaAp-LacZ	*E. coli dnaA-PAmCherry2.1* harbouring the pdnaAp-LacZ plasmid	This study
E. coli Δ3D *dnaA-PAmCherry2.1* x pdnaAp-LacZ	*E. coli* Δ3D *dnaA-PAmCherry2.1* harbouring the pdnaAp-LacZ plasmid	This study
E. coli Δ*datA dnaA-PAmCherry2.1* x pdnaAp-LacZ	*E. coli* Δ*datA dnaA-PAmCherry2.1* harbouring the pdnaAp-lacZ plasmid	This study
E. coli Δ*DARS1 dnaA-PAmCherry2.1* x pdnaAp-LacZ	*E. coli* Δ*DARS1 dnaA-PAmCherry2.1* harbouring the pdnaAp-lacZ plasmid	This study
E. coli Δ*DARS2 dnaA-PAmCherry2.1* x pdnaAp-LacZ	*E. coli* Δ*DARS2 dnaA-PAmCherry2.1* harbouring the pdnaAp-lacZ plasmid	This study

Table S2. List of plasmids used in this study. *DnaA(n1-n2)* stands for the sequence of the *dnaA* gene comprised between base pair n1 and base pair n2, counting from the G at position 1.

Plasmid	Characteristics	Source
pCas9	*repA101*(Ts), *kanR, cas9,* P_{araB}*-Red, lacI*^{q}*,* P_{trc}-sgRNA-*pMB1*	[2]
pSIJ8	*repA101*(Ts), *ampR,* P_{araB}*-Red, rhaRS,* P_{rhaB}*-flp*	[3]
pCas9_ampR	pCas9; Δ*kanR ampR*	This study
pTarget	pMB1; *aadA, pJ23119*	[2]
pTarget_dnaA-PAFP series		
pTarget_dnaA-PAmCherry2.1	pTarget; *pJ23119*-sgRNA(*dnaA*), *dnaA(209-258)-PAmCherry2.1-dnaA(313-362)*	This study
pTarget_dnaA-Dronpa2	pTarget; *pJ23119*-sgRNA(*dnaA*), *dnaA(209-258)-Dronpa2-dnaA(313-362)*	This study
pTarget_dnaA-mEos4b	pTarget; *pJ23119*-sgRNA(*dnaA*), *dnaA(209-258)-mEos4b-dnaA(313-362)*	This study
pTarget_dnaA-mMaple3	pTarget; *pJ23119*-sgRNA(*dnaA*), *dnaA(209-258)-mMaple3-dnaA(313-362)*	This study
pDnaA211-PAmCherry2.1	*repE, ori2, sopABC, cat,* $P_{LtetO-1}$*-BCD-dnaA(M426T)-PAmCherry2.1*	This study
pdnaAp-lacZ	*repA101 ampR dnaAp-lacZ*	This study

Table S3. List of oligonucleotides and chemically synthesised DNA fragments and their use in the study.

Identifier	Sequence (5'-3')	Used for
BG25452	ATGTCGGTGATCAAACCAGATATGAAGATCAAACTTCGTATGGAGGGAGCG<br>GTCAATGGGCATCCTTTCGCGATCGAGGGTGTCGGGCTGGGCAAACCCTTCG<br>AAGGGAAGCAAAGTATGGACTTAAAAGTCAAAGAGGGAGGCCCGTTACCCT<br>TTGCGTATGATATTTTGACCACAGTTTTTTGCTACGGGAATCGCGTATTTGCT<br>AAGTACCCGGAAAACATCGTCGATTACTTCAAACAGAGCTTCCCAGAAGGAT<br>ATAGTTGGGAGCGCTCAATGAACTACGAGGACGGTGGCATTTGTAATGCGA<br>CGAACGACATTACATTAGACGGCGACTGCTACATTTATGAGATCCGTTTCGA<br>CGGTGTCAACTTTCCAGCGAATGGACCTGTGATGCAAAAGCGCACTGTAAAA<br>TGGGAACCTTCAACCGAGAAGTTATATGTGCGTGACGGGGTCTTAAAAGGC<br>GATGTAAATACCGCTTTGAGTTTAGAGGGAGGTGGCCATTACCGTTGCGATT<br>TCAAAACGACTTATAAGGCAAAAAAAGTGGTACAATTACCTGATTATCACTTC<br>GTCGATCATCACATCGAGATTAAATCACATGATAAGGACTATTCTAATGTGA<br>ATCTTCACGAACATGCCGAGGCCCATAGTGAGTTACCGCGTCAAGCAAAGTA<br>A	*E. coli* codon-optimised *dronpa2.*
BG25454	ATGGTTAGTGCGATTAAACCAGATATGCGTATCAAGTTGCGTATGGAGGGA<br>AATGTTAATGGACATCACTTCGTCATTGATGGAGACGGAACTGGCAAGCCGT<br>ACGAGGGTAAGCAGACCATGGACCTGGAGGTCAAGGAGGGTGGCCCATTG<br>CCGTTCGCGTTTGATATCCTGACGACAGCTTTCCACTATGGGAATCGTGTCTT<br>CGTAAAATATCCAGATAACATCCAGGACTATTTCAAGCAGTCATTTCCCAAAG<br>GTTACTCTTGGGAACGCAGCTTAACCTTCGAAGACGGGGGGATTTGCAACGC<br>CCGCAACGACATTACTATGGAGGGAGACACGTTTTACAACAAAGTGCGTTTT<br>TATGGAACAAACTTCCCGGCGAACGGGCCTGTTATGCAGAAGAAGACTCTG<br>AAGTGGGAGCCGTCCACGGAAAAGATGTACGTGCGCGATGGGGTCTTAACT<br>GGGGATATTGAGATGGCCTTGCTGCTTGAAGGTAATGCTCACTACCGCTGCG<br>ATTTCCGTACGACCTATAAGGCAAAAGAAAAGGGGGTCAAGTTGCCAGGTG<br>CTCATTTTGTTGATCACGCGATTGAAATTTTGTCGCATGATAAGGATTACAAT<br>AAGGTTAAACTTTACGAGCATGCGGTGGCTCATAGCGGTCTTCCCGACAATG<br>CCCGTCGTTAA	*E. coli* codon-optimised *mEos4b.*
BG25455	ATGGTCTCTAAGGGAGAAGAGACGATCATGTCGGTAATCAAACCGGATATG<br>AAAATCAAGCTTCGCATGGAAGGTAACGTCAACGGTCACGCCTTTGTCATCG<br>AAGGTGAAGGCTCAGGTAAGCCATTTGAAGGTATCCAAACCATCGACTTGG<br>AAGTAAAGGAGGGTGCGCCTTTACCATTCGCATACGACATTCTTACCACCGC<br>TTTCCATTACGGAAACCGCGTGTTCACCAAGTACCCTCGCAAGATCCCTGACT<br>ACTTCAAACAGAGCTTCCCAGAGGGATATTCTTGGGAACGTAGTATGACGTA<br>CGAGGACGGGGGTATCTGCAATGCGACTAATGATATTACAATGGAAGAAGA<br>TTCGTTTATCAATAAAATTCACTTCAAAGGTACGAATTTTCCGCCCAACGGCC<br>CGGTAATGCAGAAACGTACTGTAGGTTGGGAGGTCTCGACTGAAAAGATGT<br>ATGTTCGTGACGGCGTGCTGAAGGGAGATGTTAAAATGAAGCTGCTTCTTAA<br>GGGTGGCTCCCATTATCGCTGTGATTTTCGTACCACATACAAGGTGAAGCAA<br>AAAGCAGTGAAATTGCCCAAAGCACACTTTGTCGACCATCGTATCGAGATCC<br>TTTCTCACGATAAAGATTACAACAAGGTTAAGTTGTATGAACATGCTGTAGCT<br>CGCAATTCCACAGATAGTATGGACGAGCTGTATAAATAA	*E. coli* codon-optimised *mMaple3.*
BG21364	TCAGCCTTAGTCATTATCGAC	Amplified the *dnaA* gene in *E. coli* chromosome

BG21365	GGTTTACGATGACAATGTTCTG	Amplified *dnaA* gene in all *E. coli* strains
BG23801	CGTATAATGCGCCTCCCG	Amplified Δ*datA::kan* in *E. coli* Δ*datA*
BG23802	CCGAGCCCAAACTGCAAG	Amplified Δ*datA::kan* in *E. coli* Δ*datA*
BG23803	CAGAAAATGCGGCAACCGG	Amplified Δ*DARS1::cat* in *E. coli* Δ*DARS1*
BG23804	AGTTGGGCGGGCAGGTATG	Amplified Δ*DARS1::cat* in *E. coli* Δ*DARS1*
BG23805	GTAAACCACTCTCTGCAGGG	Amplified Δ*DARS2::cat* in *E. coli* Δ*DARS2*
BG23806	GTTGGGACATGTCATGATACC	Amplified Δ*DARS2::cat* in *E. coli* Δ*DARS2*
BG36136	CGCGCCAGATGGCGACCGCGCTGGCGAAAGAGCTG	Introduced M426T in *dnaA-PAmCherry2.1*
BG36137	CGCGGTCGCCATCTGGCG	Introduced M426T in *dnaA-PAmCherry2.1*
BG35613	TACGGGCTGATGGGTAAAGC	Amplified *dnaAp* from the genome of *E. coli* MG1655
BG35614	GCGGACTCCACTCGAACAAAAG	Amplified *dnaAp* from the genome of *E. coli* MG1655
BG36203	TTTGTTCGAGTGGAGTCCGCCATGACCATGATTACGGATTCACTGGC	Amplified *lacZ* from the genome of *E. coli* MG1655
BG36204	TTATTTTTGACACCAGACCAACTG	Amplified *lacZ* from the genome of *E. coli* MG1655

Table S4. Number of DnaA boxes counted on the genome of different *E. coli* strains, as well as *Salmonella enterica.* Accession identifier NC_000913.3 refers to the wild-type *E. coli* MG1655 strain. Source data are provided as a Source Data file.

Strain accession identifier	Number of counted DnaA boxes
NC_000913.3	775
NC_002655.2	1019
NC_002695.2	997
NC_004431.1	956
NC_007946.1	879
NC_008253.1	887
NC_008563.1	871
NC_009800.1	779
NC_009801.1	887
NC_010468.1	838
NC_010473.1	778
NC_010498.1	924
NC_011353.1	1014
NC_011415.1	845
NC_011601.1	883
NC_011741.1	802
NC_011742.1	874
NC_011745.1	940
NC_011748.1	901
NC_011750.1	942
NC_011751.1	887
NC_012759.1	762
NC_012967.1	799
NC_013008.1	1006
NC_013353.1	954
NC_013361.1	1022
NC_013364.1	954
NC_013654.1	819
NC_013941.1	973
NC_017625.1	773
NC_017626.1	932
NC_017628.1	888
NC_017631.1	936
NC_017632.1	873
NC_017633.1	840
NC_017634.1	800
NC_017638.1	771
NC_017641.1	889
NC_017646.1	964
NC_017651.1	912
NC_017652.1	912
NC_017656.1	958

NC_017906.1	976
NC_018650.1	935
NC_018658.1	940
NC_018661.1	950
NC_020163.1	816
NC_022648.1	921
NZ_CP006027.1	1018
NZ_CP006262.1	990
NZ_CP007133.1	990
NZ_CP007136.1	1018
NZ_CP007265.1	783
NZ_CP008805.1	1007
NZ_CP010344.1	832
NZ_HG941718.1	905

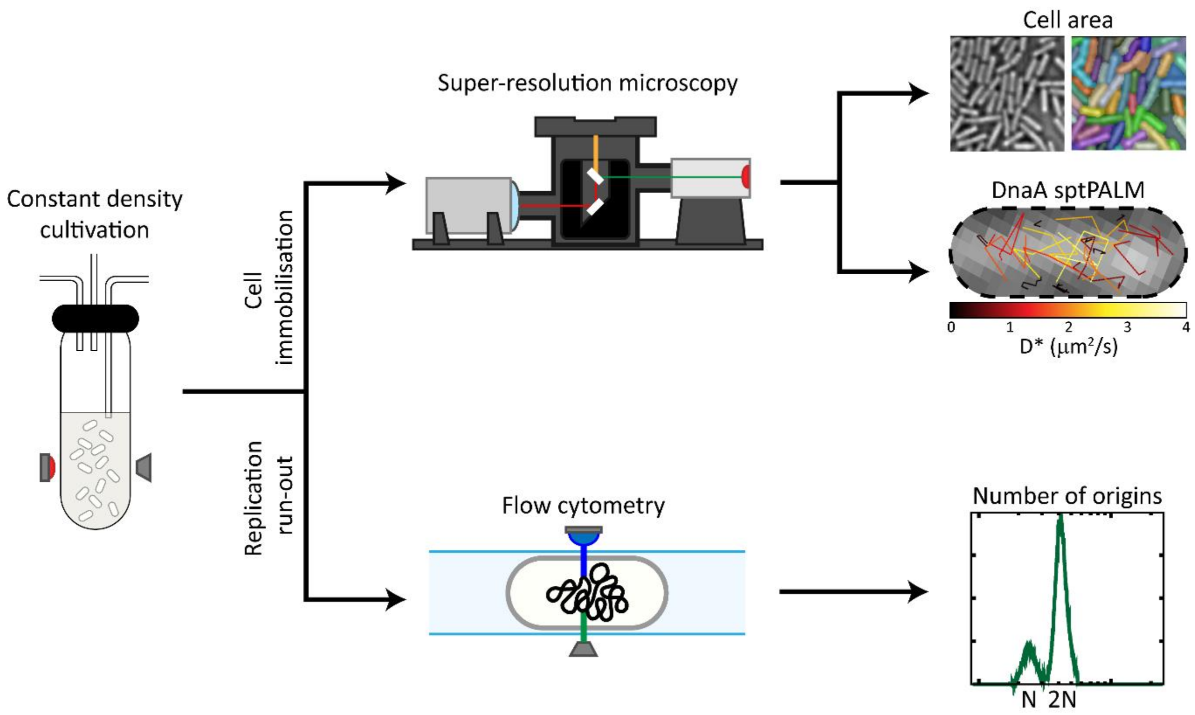

Supplementary Figure 1. Experimental pipeline for the single-molecule characterisation of DnaA titration, related to Figure 1. After an initial growth phase in turbidostat with three different type of media, leading to either slow, intermediate or fast growth regime, cells are processed accordingly to the intended measurements. A first sample (10 mL) is collected, washed in PBS and immobilised on agarose slabs for follow-up microscopy. From the sptPALM imaging, cell area measurements and diffusion coefficients of DnaA tracks were collected. In parallel, replication run-out was performed on the remainder of the cellular culture, by providing rifampicin (150 μg/mL final concentration) and cephalexin (15 μg/mL final concentration) and leave incubating for additional 6 h 30 min at cultivation temperature. A sample of synchronised cells (3 mL) is then collected, fixated and permeabilised in 70% ethanol and then supplemented with PicoGreen before being subjected to flow cytometry for estimating the number of origins of the population.

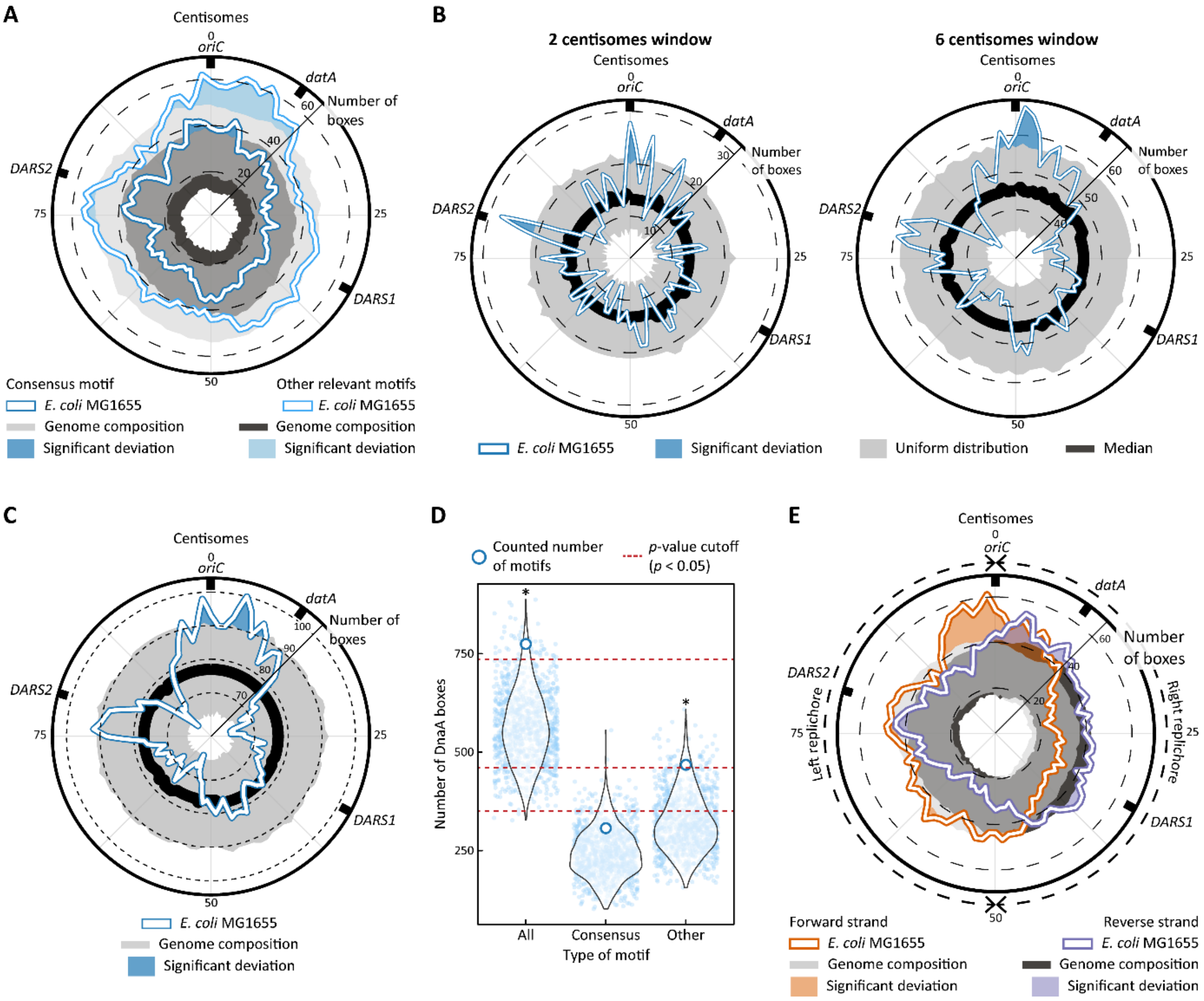

Supplementary Figure 2. Computational identification of enrichment of DnaA boxes on the chromosome of *E. coli*, related to Figure 2. In all plots, the *E. coli* chromosome is represented as a circle, with distance units in centisomes. The radius of the circle represents the number of boxes counted in the genome of *E. coli* MG1655. **A)** Both the counted consensus DnaA boxes (TTWTNCACA, dark blue) and other sequences (HHMTHCWVH, light blue) are preferentially accumulated towards *oriC,* as observed when compared to the expected range from genome composition. **B)** Lowering the window of plotted DnaA boxes counts to either 2 centisomes (left) or 6 centisomes (right) maintains the significant enrichment of DnaA boxes towards *oriC*. **C)** Even when excluding the region of *oriC* from the analysis, DnaA boxes are still enriched between centisomes 98 and 5 of the *E. coli* chromosome. **D)** The total amount of counted DnaA boxes (blue empty dot) is significantly higher than what expected from genome composition (red line indicates threshold of $p < 0.05$). Following the same analysis, the number of DnaA boxes matching the consensus motif is not enriched, but the number of boxes with other type of motifs (blue empty dots) is enriched. The range expected from genome composition was obtained by permutating sequences 1000 times and counting the instances of each permutated sequence (blue filled dot). **E)** The orange line indicates the boxes counted on the forward strand of *E. coli* MG1655 genome, with coloured areas indicating areas where the count is higher than the expected range from genome composition. Similarly, the purple line and purple area indicate the boxes counted on the reverse strand and enriched areas, respectively. The two replichores are indicated outside: in the right replichore, the reverse strand is the lagging strand during DNA replication, whereas the opposite is true for the left replichore (forward strand is the lagging strand). Source data are provided as a Source Data file.

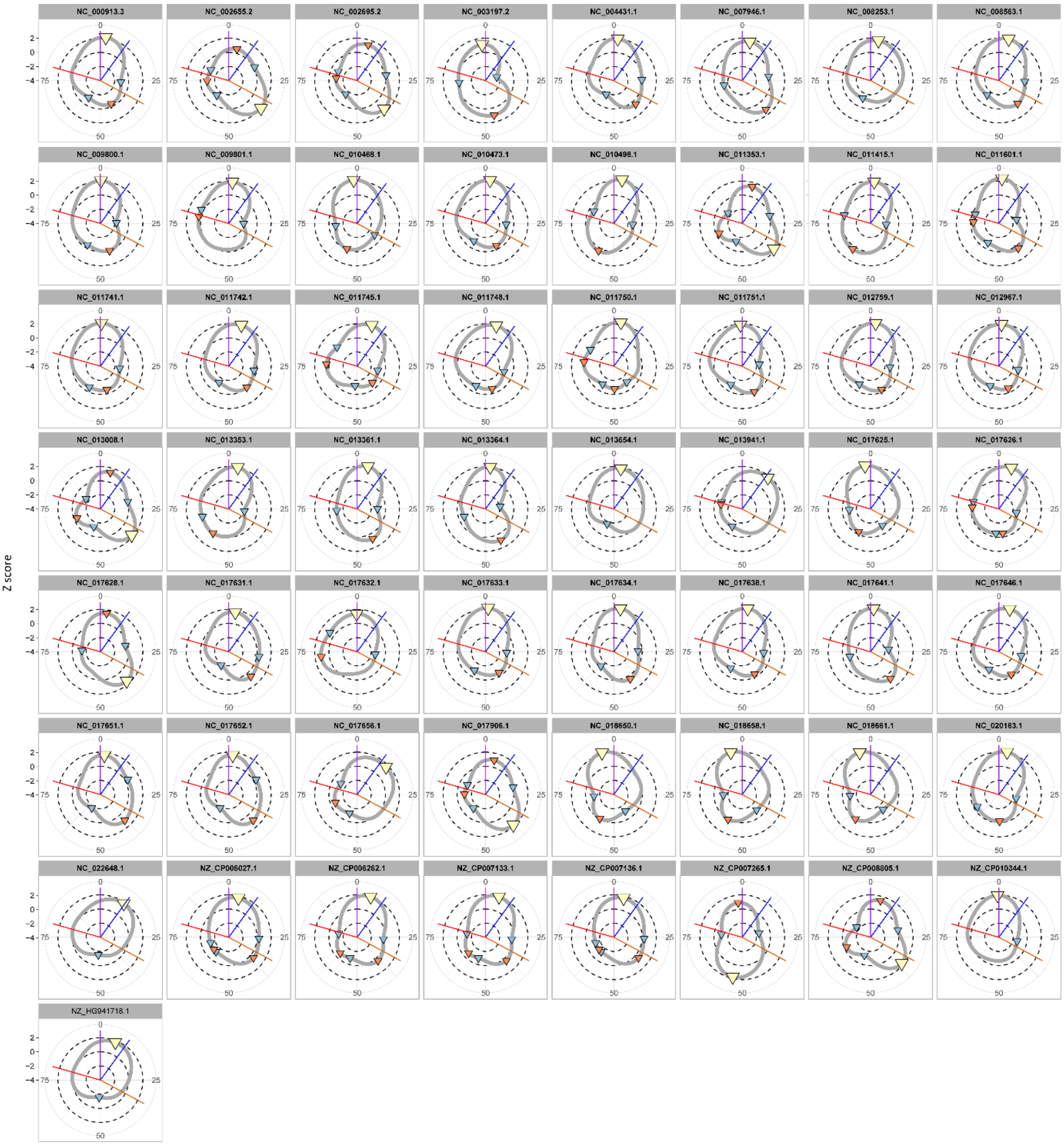

Supplementary Figure 3. Individual density plot of each *E. coli* strain with an available sequenced genome, related to Figure 2B. The accession number of each genome is on top of the related graph. Yellow triangles indicate the global maximum, red triangles indicate local maxima and blue triangles indicate local minima. The purple line indicates *oriC*, the blue line indicates *datA,* the orange line indicate *DARS1* and the red line *DARS2.* Source data are provided as a Source Data file.

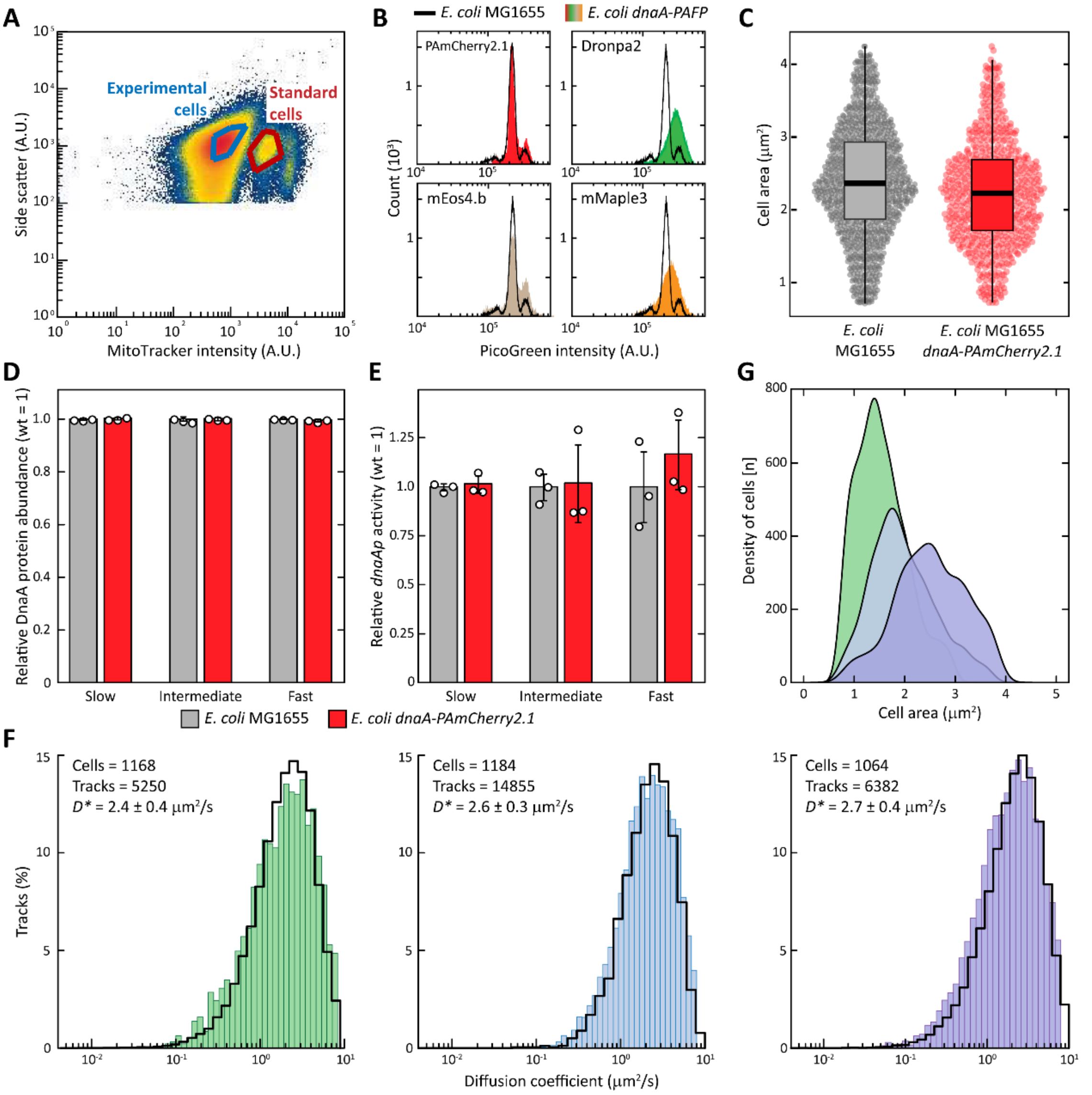

Supplementary Figure 4. Characterisation of the mutant *E. coli* MG1655 *dnaA-PAmCherry2.1*, related to Figure 3. A) Separation of reference and experimental cells by gating for MitoTracker intensity. Cells with a higher far-red emission, derived from the MitoTracker™ dye, were gated as reference cells, whereas the other population was gated as experimental cells. **B)** DNA content of *E. coli* strains carrying fusions of DnaA with PAmCherry2.1, Dronpa2, mEos4.b or mMaple3 when compared to the wild-type *E. coli* MG1655 reference (black line). All strains were grown in LB prior to rifampicin run-out and flow cytometry. At least 10000 cells have been collected for each condition and replicate. **C)** Area measurements of *E. coli* MG1655 and *E. coli* MG1655 *dnaA-PAmCherry2.1* grown in M9 medium, supplemented with 0.4% glucose and 1x RPMI amino acids. Box plots indicate median (middle line), 25th and 75th percentile (box) and 5th and 95th percentile (whiskers). **D)** Expression of DnaA in *E. coli* MG1655 *dnaA-PAmCherry2.1* (red bars) and *E. coli* MG1655 (grey bars) in either slow, intermediate and fast growth regime. The measured mass spectrometric protein intensity of DnaA was normalised for the average signal of the wild-type strain. Data are presented as mean values ± SD, n = 3 biologically independent replicates. **E)** *dnaAp* promoter activity in *E. coli* MG1655 *dnaA-PAmCherry2.1* (red bars) and *E. coli* MG1655 (grey bars) in either slow, intermediate and fast growth regime. The activity of the LacZ β-galactosidase was normalised for the average value of the wild-type strain in the same growth condition. Data are presented as mean values ± SD, n = 3 biologically independent replicates. **F)** Diffusion coefficient histograms of DnaA211 obtained from sptPALM with cells growing in slow (green), intermediate (blue) or fast (purple) growth regime. Histograms are fitted (black line) with a theoretical description of 250,000 particles in a static species, yielding an average diffusion coefficient of 2.6 ± 0.2 μm²/s. Individual diffusion coefficients are shown in the upper left corner in each growth regime. **G)** Distribution of cellular area of *E. coli* MG1655 *dnaA-PAmCherry2.1* cells grown in either slow (green), intermediate (blue) or fast growth regime (purple) and used for studying the mobility of DnaA across the cell cycle. Source data are provided as a Source Data file.

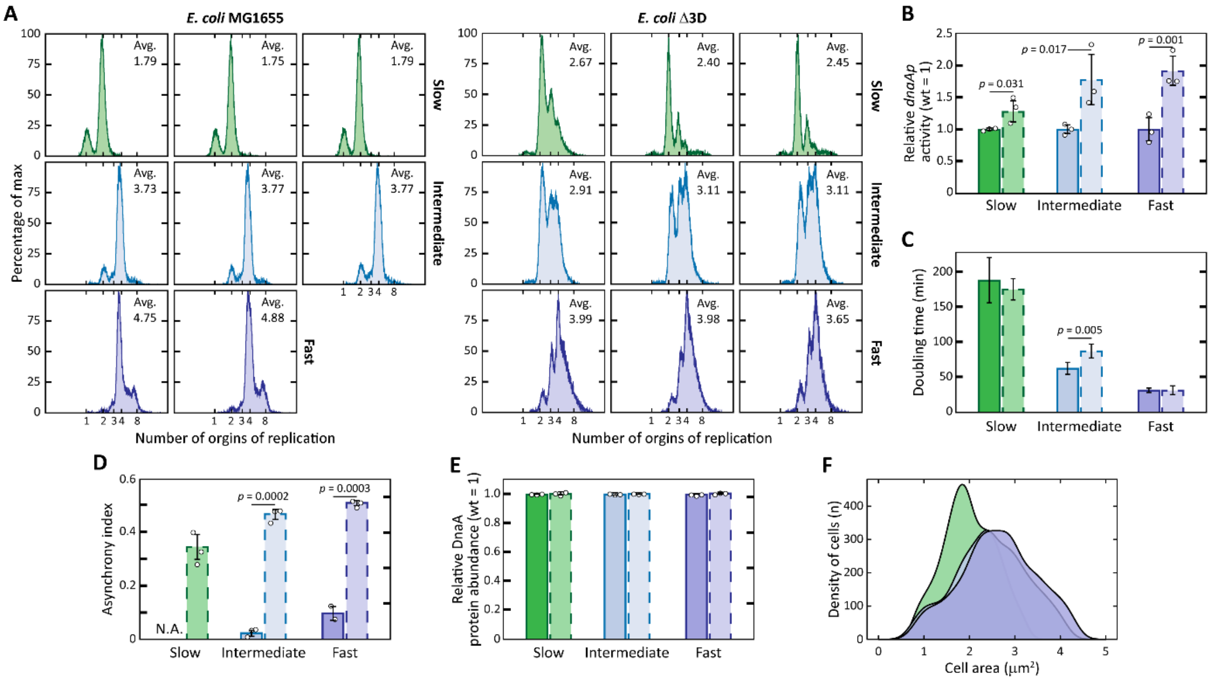

Supplementary Figure 5. Cellular parameters used in the study of *E. coli* Δ3D *dnaA-PAmCherry2.1*, related to Figure 4. In each panel, slow growth regime is indicated in green, intermediate in blue and fast in purple. **A)** Raw data of every replicate of DNA content measurements via flow cytometry of either *E. coli* MG1655 *dnaA-PAmCherry2.1* (left) and *E. coli* Δ3D *dnaA-PAmCherry2.1* (right) in different growth regimes. Average number of origin of replication for each condition is indicated for each replicate. At least 10000 cells have been collected for each condition and replicate. **B)** *dnaAp* promoter activity in the mutant *E. coli* Δ3D *dnaA-PAmCherry2.1* (dashed outlines) compared to the wild-type *E. coli* MG1655 *dnaA-PAmCherry2.1* (continuous outline). The activity of the LacZ β-galactosidase of *E. coli* Δ3D *dnaA-PAmCherry2.1* was normalised for the average value of the wild-type strain in the same growth condition. Data are presented as mean values ± SD, *n* = 3 biologically independent replicates. *P*-values are calculated by a two-sided T test. **C)** Doubling times of *E. coli* MG1655 *dnaA-PAmCherry2.1* (continuous outline) and *E. coli* Δ3D *dnaA-PAmCherry2.1* (dashed outline). Data are presented as mean values ± SD, *n* = 3 biologically independent replicates. *P*-values are calculated by a two-sided T test. **D)** Asynchrony index of either *E. coli* MG1655 *dnaA-PAmCherry2.1* or *E. coli* Δ3D *dnaA-PAmCherry2.1.* This value was obtained as Asynchrony index = $\frac{f3+f5+f6+f7}{f2+f4+f8}$, where f_X is the number of cells with X origins of replication[4], as estimated from the replication run-out experiments. Asynchrony index cannot be obtained for *E. coli* MG1655 during slow growth, as cells only cycled between 1 and 2 origins of replication. Data are presented as mean values ± SD, *n* = 3 biologically independent replicates. *P*-values are calculated by a two-sided T test. **E)** Expression of DnaA in *E. coli* Δ3D *dnaA-PAmCherry2.1* (dashed outlines) compared to the wild-type *E. coli* MG1655 *dnaA-PAmCherry2.1* (continuous outline). The measured mass spectrometric protein intensity of DnaA was normalised for the average signal of the wild-type strain. Data are presented as mean values ± SD, *n* = 3 biologically independent replicates. **F)** Distribution of cellular area of *E. coli* Δ3D *dnaA-PAmCherry2.1* cells in different growth regimes used for studying the mobility of DnaA across the cell cycle. Source data are provided as a Source Data file.

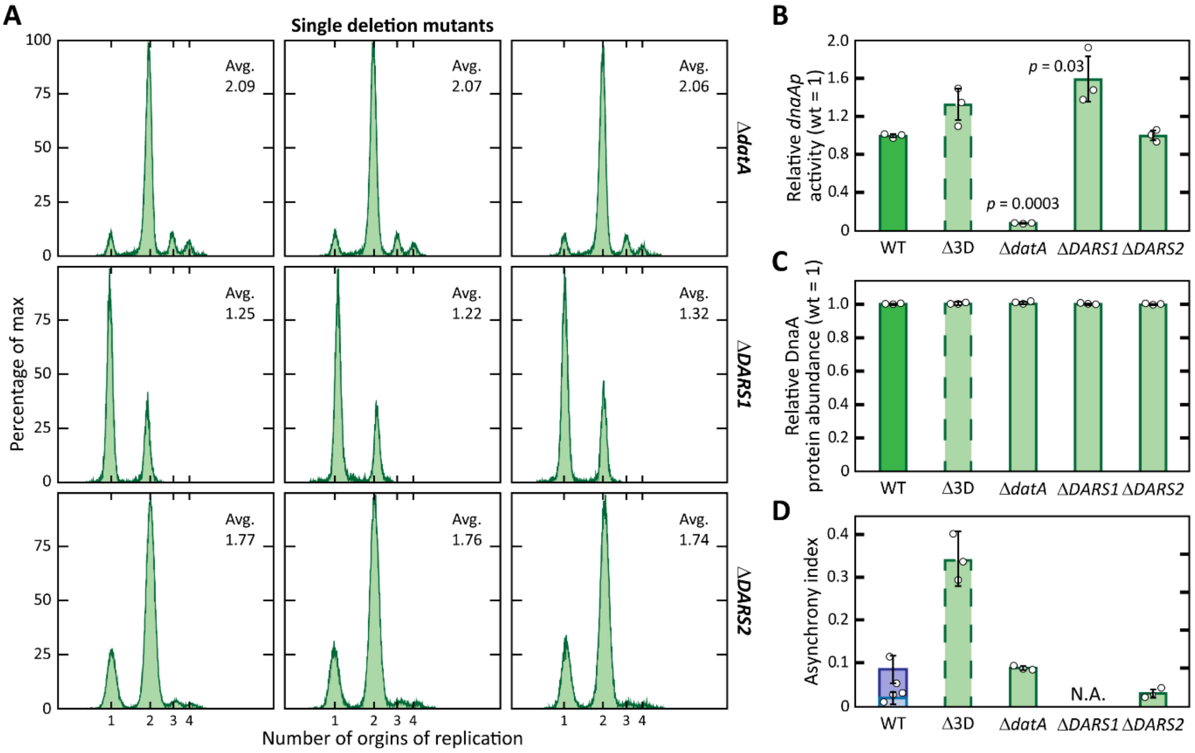

Supplementary Figure 6. Cellular parameters used in the study of *E. coli* Δ*datA,* Δ*DARS1* and Δ*DARS2*, carrying the *dnaA-PAmCherry2.1* locus, related to Figure 5. A) Raw data of every replicate of DNA content measurements via flow cytometry of either *E. coli* Δ*datA dnaA-PAmCherry2.1* (top), *E. coli* Δ*DARS1 dnaA-PAmCherry2.1* (middle) and *E. coli* Δ*DARS2 dnaA-PAmCherry2.1* (bottom) in slow growth regime. Average number of origin of replication for each condition is indicated for each replicate. At least 10000 cells have been collected for each condition and replicate. **B)** *dnaAp* promoter activity in the wild-type *E. coli* MG1655 *dnaA-PAmCherry2.1* and the mutant strains *E. coli* Δ3D *dnaA-PAmCherry2.1, E. coli* Δ*datA dnaA-PAmCherry2.1, E. coli* Δ*DARS1 dnaA-PAmCherry2.1* and *E. coli* Δ*DARS2 dnaA-PAmCherry2.1*. The activity of the LacZ β-galactosidase was normalised for the average value of the wild-type strain. Data are presented as mean values ± SD, *n* = 3 biologically independent replicates. *P*-values are calculated by a two-sided T test, always comparing with the wild-type. **C)** Expression of DnaA in the wild-type *E. coli* MG1655 *dnaA-PAmCherry2.1* and the mutant strains *E. coli* Δ3D *dnaA-PAmCherry2.1, E. coli* Δ*datA dnaA-PAmCherry2.1, E. coli* Δ*DARS1 dnaA-PAmCherry2.1* and *E. coli* Δ*DARS2 dnaA-PAmCherry2.1*. The measured mass spectrometric protein intensity of DnaA (LFQ intensity) was normalised for the signal of the wild-type strain. Data are presented as mean values ± SD, *n* = 3 biologically independent replicates. **D)** Asynchrony index of either *E. coli* Δ*datA dnaA-PAmCherry2.1*, *E. coli* Δ*DARS1 dnaA-PAmCherry2.1* and *E. coli* Δ*DARS2 dnaA-PAmCherry2.1*, with *E. coli* MG1655 and *E. coli* Δ3D for comparison. For *E. coli* MG1655, the values obtained for cells grown in intermediate and fast growth regime are provided, as the asynchrony index cannot be calculated for slow growth. Data are presented as mean values ± SD, *n* = 3 biologically independent replicates. Source data are provided as a Source Data file.

Supplementary References

1. Frimodt-Møller, J., Charbon, G., Krogfelt, K. A. & Løbner-Olesen, A. Control regions for chromosome replication are conserved with respect to sequence and location among *Escherichia coli* strains. *Front. Microbiol.* **6**, (2015).

2. Jiang, Y. *et al.* Multigene editing in the *Escherichia coli* genome via the CRISPR-Cas9 system. *Appl. Environ. Microbiol.* **81**, 2506–2514 (2015).

3. Jensen, S. I., Lennen, R. M., Herrgård, M. J. & Nielsen, A. T. Seven gene deletions in seven days: Fast generation of *Escherichia coli* strains tolerant to acetate and osmotic stress. *Sci. Rep.* **5**, (2015).

4. Olsson, J. A., Nordström, K., Hjort, K. & Dasgupta, S. Eclipse–Synchrony Relationship in *Escherichia coli* Strains with Mutations Affecting Sequestration, Initiation of Replication and Superhelicity of the Bacterial Chromosome. *J. Mol. Biol.* **334**, 919–931 (2003).
