# Supplementary material for: The Escherichia coli replication initiator DnaA is titrated on the chromosome
Source: Nat Commun. 2025 Aug 21;16:7813. doi: 10.1038/s41467-025-63147-1 (PMC12371093; doi:10.1038/s41467-025-63147-1)
Supplement: Supplementary file 2 — Description of Additional Supplementary Files [file 41467_2025_63147_MOESM2_ESM.pdf]

Description of Additional Supplementary Files:

Supplementary Movie 1: Diffusion of individual DnaA-PAmCherry2.1 proteins inside the cell. Cumulative overlay of tracks are plotted on top of a brightfield image of *E. coli* cells. Each track is coloured differently. The video shows a small part of a normal field of view.
