# Supplementary material for: The Escherichia coli replication initiator DnaA is titrated on the chromosome
Source: Nat Commun. 2025 Aug 21;16:7813. doi: 10.1038/s41467-025-63147-1 (PMC12371093; doi:10.1038/s41467-025-63147-1)
Supplement: Supplementary file 4 — Reporting Summary [file 41467_2025_63147_MOESM4_ESM.pdf]

nature portfolio

Corresponding author(s): Lorenzo Olivi, Johannes Hohlbein, Raymond H.J. Staals

Last updated by author(s): Jul 28, 2025

Reporting Summary

Nature Portfolio wishes to improve the reproducibility of the work that we publish. This form provides structure for consistency and transparency in reporting. For further information on Nature Portfolio policies, see our Editorial Policies and the Editorial Policy Checklist.

Statistics

For all statistical analyses, confirm that the following items are present in the figure legend, table legend, main text, or Methods section.

n/a	Confirmed	
☐	☒	The exact sample size (*n*) for each experimental group/condition, given as a discrete number and unit of measurement
☐	☒	A statement on whether measurements were taken from distinct samples or whether the same sample was measured repeatedly
☐	☒	The statistical test(s) used AND whether they are one- or two-sided<br>*Only common tests should be described solely by name; describe more complex techniques in the Methods section.*
☒	☐	A description of all covariates tested
☒	☐	A description of any assumptions or corrections, such as tests of normality and adjustment for multiple comparisons
☐	☒	A full description of the statistical parameters including central tendency (e.g. means) or other basic estimates (e.g. regression coefficient) AND variation (e.g. standard deviation) or associated estimates of uncertainty (e.g. confidence intervals)
☐	☒	For null hypothesis testing, the test statistic (e.g. *F*, *t*, *r*) with confidence intervals, effect sizes, degrees of freedom and *P* value noted<br>*Give P values as exact values whenever suitable.*
☒	☐	For Bayesian analysis, information on the choice of priors and Markov chain Monte Carlo settings
☒	☐	For hierarchical and complex designs, identification of the appropriate level for tests and full reporting of outcomes
☒	☐	Estimates of effect sizes (e.g. Cohen's *d*, Pearson's *r*), indicating how they were calculated

Our web collection on statistics for biologists contains articles on many of the points above.

Software and code

Policy information about availability of computer code

Data collection	The genomes used in our bioinformatics analysis were download from RefSeq. A complete list of accession numbers is provided in the Source Data file.<br>The data on the average number of oriC was obtained through flow cytometry using the Attune NxT Flow Cytometer (Invitrogen).<br>Imaging data was obtained using the miCube super-resolution microscope.<br>The proteomics data was obtained using a Vanquish Neo UHPLC (ThermoFisher Scientific) coupled with a Orbitrap Exploris 480 mass spectrometer (ThermoFisher Scientific).<br>Data on the LacZ activity was obtained using a Synergy H1 microplate reader (BioTek) and the Gen5 software (BioTek).
Data analysis	Genomes were analysed with a custom-made Python pipeline to obtain the data on DnaA boxes location and positional enrichment. The version used in the study is available for download from the Zenodo repository (https://doi.org/10.5281/zenodo.13939193). Any updates can be found in the GitHub repository (https://github.com/stephkoest/Ecoli_titration). Both hyperlinks are listed in the Code Availability section of the manuscript.<br>Attune Flow Cytometer software was used to analyse the flow cytometry data.<br>Imaging data was first processed through the ImageJ/FIJI plugins Interactive Watershed (http://imagej.net/Interactive_Watershed), FTM2 (https://github.com/HohlbeinLab/FTM2) and ThunderSTORM. The output of the different plugins were then used as input in the custom-made MATLAB pipeline to generate single-particle tracking data. All the scripts that are part of the pipeline are available for download from the Zenodo repository (https://doi.org/10.5281/zenodo.13939193).<br>The proteomics data was analysed using the DIA-NN pipeline using the Uniprot reference fasta file for E. coli MG1655 K-12 (UP000000625).

For manuscripts utilizing custom algorithms or software that are central to the research but not yet described in published literature, software must be made available to editors and reviewers. We strongly encourage code deposition in a community repository (e.g. GitHub). See the Nature Portfolio guidelines for submitting code & software for further information.

Data

Policy information about availability of data

All manuscripts must include a data availability statement. This statement should provide the following information, where applicable:

- Accession codes, unique identifiers, or web links for publicly available datasets
- A description of any restrictions on data availability
- For clinical datasets or third party data, please ensure that the statement adheres to our policy

The data supporting our finding is available as Source Data with the manuscript. Bacterial strains used in the research is available upon request to the corresponding authors. Mass spectrometric raw data and DIA-NN output files are is available through the ProteomeXchange Consortium via the PRIDE partner repository (accession number PXD064841; direct link https://proteomecentral.proteomexchange.org/cgi/GetDataset?ID=PXD064841).

Research involving human participants, their data, or biological material

Policy information about studies with human participants or human data. See also policy information about sex, gender (identity/presentation), and sexual orientation and race, ethnicity and racism.

Reporting on sex and gender	Not applicable.
Reporting on race, ethnicity, or other socially relevant groupings	Not applicable.
Population characteristics	Not applicable.
Recruitment	Not applicable.
Ethics oversight	Not applicable.

Note that full information on the approval of the study protocol must also be provided in the manuscript.

Field-specific reporting

Please select the one below that is the best fit for your research. If you are not sure, read the appropriate sections before making your selection.

☒ Life sciences ☐ Behavioural & social sciences ☐ Ecological, evolutionary & environmental sciences

For a reference copy of the document with all sections, see nature.com/documents/nr-reporting-summary-flat.pdf

Life sciences study design

All studies must disclose on these points even when the disclosure is negative.

Sample size	All data describe in the study is a mean of three indipendent biological replicates, obtained by culturing the strains three separate times in three separate turbidostat reactors. For single-particle tracking, we considered 5000 tracks to be the minimum number of total tracks to analyse to obtain reliable diffusion coefficient distributions. For flow cytometry, we considered 10000 cells as the minimum number to obtain reliable informations on DNA content. In all cases, the chosen number of independent biological replicates, protein tracks and flow cytometry events are considered as the standard to obtain statistically relevant information on biological systems in the respective fields.
Data exclusions	We did not exclude any of the obtained biological replicates.
Replication	All attempts of replication were successful.
Randomization	Data generated from biological replicates of the same conditions were grouped together during analysis to obtain statistically relevant information on the behaviour of the specific experimental condition. This strategy is the standard in the field. Therefore, no randomisation strategy was applied in the present study.
Blinding	We predetermined a pipeline for sampling collection and data collection, as well as data analysis at the beginning of the study and followed it for all samples obtained throughout it. As such, no blinding strategy was necessary.

Reporting for specific materials, systems and methods

We require information from authors about some types of materials, experimental systems and methods used in many studies. Here, indicate whether each material, system or method listed is relevant to your study. If you are not sure if a list item applies to your research, read the appropriate section before selecting a response.

Materials & experimental systems

n/a	Involved in the study
☒	☐ Antibodies
☒	☐ Eukaryotic cell lines
☒	☐ Palaeontology and archaeology
☒	☐ Animals and other organisms
☒	☐ Clinical data
☒	☐ Dual use research of concern
☒	☐ Plants

Methods

n/a	Involved in the study
☒	☐ ChIP-seq
☐	☒ Flow cytometry
☒	☐ MRI-based neuroimaging

Plants

Seed stocks	Not applicable.
Novel plant genotypes	Not applicable.
Authentication	Not applicable.

Flow Cytometry

Plots

Confirm that:

☒ The axis labels state the marker and fluorochrome used (e.g. CD4-FITC).

☒ The axis scales are clearly visible. Include numbers along axes only for bottom left plot of group (a 'group' is an analysis of identical markers).

☒ All plots are contour plots with outliers or pseudocolor plots.

☒ A numerical value for number of cells or percentage (with statistics) is provided.

Methodology

Sample preparation	2 mL of cells from the turbidostat reactor were added to 18 mL of 70% ethanol for fixation. Cells were left in ethanol for at least 12 hours, after which they were washed once to remove traces of medium and ethanol. Cells were then resuspended in PBS and incubated with PicoGreen and MitoTracker (for reference cells). Additional details are available in the Methods section of the manuscript.
Instrument	Attune NxT Flow Cytometer (Invitrogen).
Software	Attune Flow Cytometer Software (Invitrogen).
Cell population abundance	At least 10000 cell events were collected for each replicate. This number refers to the events considered as cells (positive to PicoGreen and eventually to MitoTracker).
Gating strategy	PicoGreen staining was used to distinguish bacterial cells from other particles or instrument noise. Additionally, reference cells were differentiated from experimental cell using the emission of the MitoTracker Deep Red FM dye (see Figure S3A in Supplementary Information to the manuscript). Cells positive to both PicoGreen and MitoTracker Deep Red FM were used as reference cells to obtain the PicoGreen intensity related to 2 copies of E. coli chromosomes. Cells only positive to PicoGreen were used as experimental cells to obtain the average number of oriC in different growth conditions and different genetic backgrounds.

☒ Tick this box to confirm that a figure exemplifying the gating strategy is provided in the Supplementary Information.
